# Design and Development of a Public AI Referee Assistance System Based on Harmony OS Platform

**DOI:** 10.3390/s25072127

**Published:** 2025-03-27

**Authors:** Jingjing Zhao, Chang Zhu, Bo Leng, Jiantao Qi

**Affiliations:** 1 Physical Education Teaching Department, China University of Petroleum (East China), Qingdao 266580, China; zhaojj@upc.edu.cn; 2 School of Information and Communication Engineering (SICE), University of Electronic Science and Technology of China, Chengdu 611731, China; 202422010912@std.uestc.edu.cn; 3 College of Education, Beijing Sports University, Beijing 100091, China; lbihh@sina.com; 4 College of New Energy, China University of Petroleum (East China), Qingdao 266580, China

**Keywords:** AI referee, object detection, motion monitoring, sports, intelligent identification

## Abstract

The Hawkeye system was regarded as a successful and effective referee assistant in commercial applications. However, the high hardware investment and strong professionalism set up a physical hurdle in public application. In this sense, one AI referee assistance system is a small-scale system developed under the guidance of computer vision theory and technology, using deep learning-based object detection algorithms and computer graphics-related knowledge. Its main function is to use computer vision technology to make correct and fair judgments on controversial decisions in badminton matches. The AI referee assistance system uses the Harmony OS platform to build a frontend mini-program and uses the YOLOV5 algorithm to detect and track corresponding targets, ultimately achieving judgments both inside and outside the bounds as well as serving violations. In this study, it was found that the object detection model trained in this study performed well, with a prediction accuracy of up to 99%. For posture recognition, this study also utilizes the relatively mature MediaPipe algorithm, which ensures high accuracy of the entire intelligent referee system’s ruling and meets the needs of current students. This helps college students better participate in badminton training and competitions, further improving their enthusiasm for participating in sports and, to some extent, solving the problem of scarce referee resources.

## 1. Introduction

Badminton is a flexible, versatile, fast, and slow, cross-net hitting sport that is deeply loved by college students. However, there is a lack of sideline referees on the badminton court for college students, resulting in a lack of a fair referee system for their off-class practice. In recent years, the combination of artificial intelligence and action video analysis has enabled the implementation of artificial intelligence referee systems [1,2,3,4,5]. As a result of interdisciplinary collaboration and universal technology, artificial intelligence technology has formed a complex technical system network along with related technologies and applications upstream and downstream.

The operating systems (OS) of mobile devices such as smartphones and tablets are mainly dominated by the iOS mobile operating system developed by Apple and the Android operating system developed by Google [6,7]. On 9 August 2019, the Huawei Developer Conference launched HarmonyOS, a microkernel-based, full scenario, and multi-terminal compatible HarmonyOS system [8]. The HarmonyOS system is the “brain” that manages computer hardware resources and is an important carrier for allocating hardware resources and implementing application software functions. Through the interconnection of various intelligent terminal devices, the compatibility problem of different attribute terminal devices has been overcome. The biggest feature of the HarmonyOS system is the ability to achieve interconnectivity between mobile phones and other smart devices, which can create many new application scenarios and great appeal for smart teaching and student teaching experience [9,10,11].

Artificial intelligence (AI) is one of the popular technologies in current online teaching [8,12,13,14]. AI refereeing is based on computer vision and studies how computers obtain high-level understanding from images or videos. In other words, computer vision is the process of using computers to mimic the visual system of living organisms. Image data are a necessary input for computer vision, including images, video sequences, or multidimensional data scanned by 3D scanners. In April 2019, a referee assistance system was officially put into use at the Stuttgart Gymnastics World Championships in Germany. The system uses AI 3D sensing technology and can serve as an auxiliary application for determining gymnastics difficulty scores.

In some sports, such as tennis, the Hawkeye system has become a part of the refereeing process. Hawkeye system was developed by engineers from Roke Manor Research Limited in Ramsey (Hampshire, UK, in 2001) [15,16,17,18,19]. This patent is held by Dr. Paul Hawkins and David Shirley. Generally, the price only for the venue and facilities of such a system can reach up to 50,000 dollars. In addition, the professional training, operations, and maintenance set up a hurdle for the public application. In this sense, one AI referee assistance system employed in this study uses the Harmony OS platform to build a frontend mini-program and uses the YOLOV5 algorithm to detect and track corresponding targets, ultimately achieving judgments on both inside and outside the bounds as well as serving violations [20,21,22].

This research is inspired by the international badminton competition eagle eye judgment system, but due to the high equipment requirements of the badminton eagle eye judgment system, which requires high-speed and high-frequency cameras, and the application scenario of international competitions for professional athletes, the ball speed can exceed 300 km/h. However, compared to current university campuses and the general social situation, the ball speed is generally much lower than that of professional athletes. So, our innovation team has renovated the existing Eagle Eye system with the primary purpose of meeting the needs of school badminton teaching, facilitating students to accurately determine the landing point of the ball during extracurricular practice and entertainment competitions, and making it easier for students to make accurate judgments on the inside and outside of each ball. This study has achieved a deep integration of deep learning and object detection, utilizing knowledge from various fields such as computer vision, computer graphics, and neural networks. The referee system we proposed, as well as the target detection ball path tactical recognition and action correction model we designed, are the first combination of deep learning, neural networks, and badminton. Our designed online badminton teaching system has great practical value in university badminton teaching and even physical education teaching.

This paper highlights the following three points. (1) For the first time, the Harmony OS platform and YOLOv5 algorithm have been combined to achieve a lightweight and low-cost AI referee system, significantly reducing hardware barriers. (2) Integrating MediaPipe pose recognition and object detection, a new method for service violation penalty is proposed to fill the gap in the field of badminton serve violation judgment using machine vision. (3) Develop a real-time referee system suitable for nonprofessional scenarios and solve the problem of real-time detection of high-speed balls (below 300 km/h) through optimized algorithms and multi-camera collaboration.

## 2. Materials and Methods

**Figure 1:**
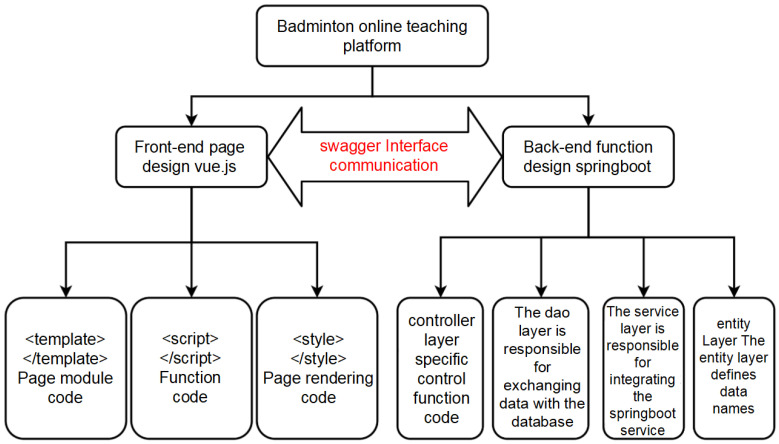
Design idea of a badminton online teaching platform.

**Figure 2:**
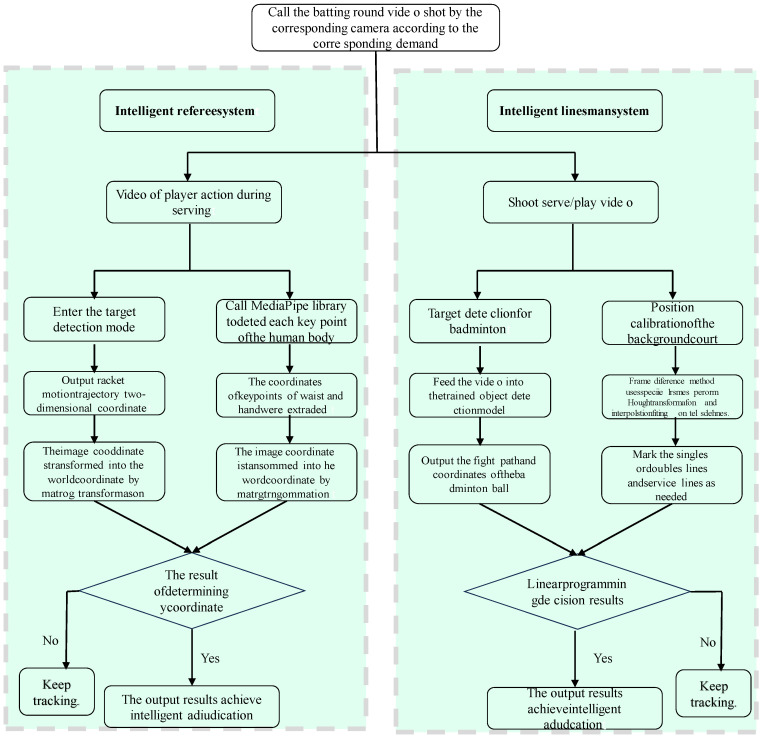
Design route of a judge system based on deep learning.

The main body of the AI referee assistance system based on the Harmony OS platform was named a “badminton online teaching platform”. It is divided into two parts, as shown in Figure 1. Firstly, the construction and improvement in the online badminton teaching platform. Secondly, the design and development of the AI referee assistance system based on deep learning. The overall design diagrams of the two systems are shown in Figure 1 and Figure 2. This study employed SpringBoot to build the entire backend framework for web pages, Vue to develop the frontend using the uni app framework for mini-programs and utilizes AJAX technology and Swagger 2.0 to achieve frontend and backend separation. In addition, MySQL 8.0 was used as the database and combined with Navicat 13 visualization tools for database design.

The reasons for choosing YOLOv5:

(1) Lightweight and real-time performance: YOLOv5 has a small model size (only 14 MB for FP16), fast inference speed (up to 30FPS on Harmony OS devices), and is suitable for mobile deployment.
(2) Multi-scale detection capability: In response to the high-speed motion characteristics of badminton, YOLOv5’s FPN + PAN structure can effectively capture small target trajectories.
(3) Open-source community support: Rich pre-trained models and transfer learning tools to accelerate model iteration.

This study tracks and records the path of the ball and displays graphical images of the actually recorded path, using a high-speed camera to simultaneously capture basic data of the badminton flight trajectory from different angles. Then, through computer calculations, three-dimensional images from these data are generated. Finally, using real-time imaging technology, the badminton’s movement route and landing point are clearly displayed on a large screen. Intelligent judgment of whether the server crosses the serving line.

In this study, the real-time images required the following:

Rotation angle: covering common shooting angle offsets in student competitions (such as handheld shaking of mobile phones);

Brightness adjustment: Simulate indoor and outdoor lighting differences (such as venue lighting and natural light);

Learning rate (0.001): determined through grid search to avoid model oscillation.

### 2.1. Design and Implementation of Front End Pages

**Figure 3:**
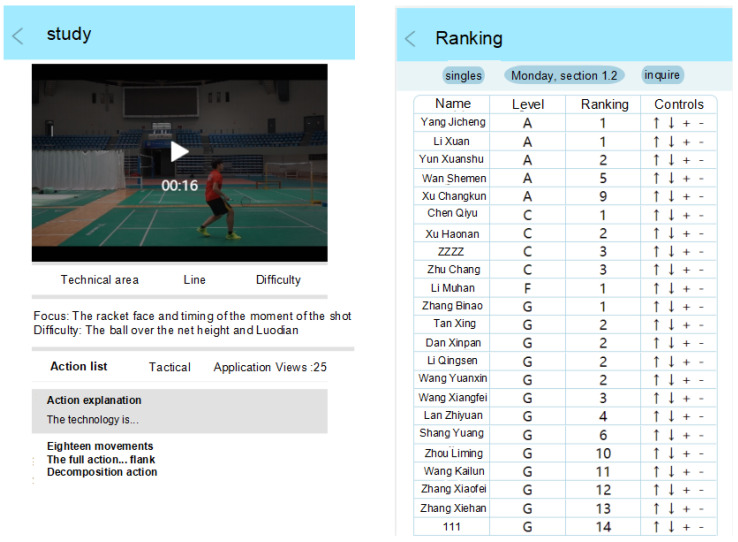
Frontend page design display.

The front page is written in Vue language and quickly built using the Uni app framework. Our research group included three student volunteers. Mr. Muhan Li is responsible for the graphic design of the page. Zhu Chang and Zhang Yifan are responsible for implementing the design using the Javascript language. The specific functional pages are shown in Figure 3. In this study, the canny algorithm was used to extract target contours, and the Hough transform interpolation fitting method was employed to fit straight line segments. Frame difference method tracks and detects target objects.

### 2.2. Design and Construction of Backend Database

**Figure 4:**
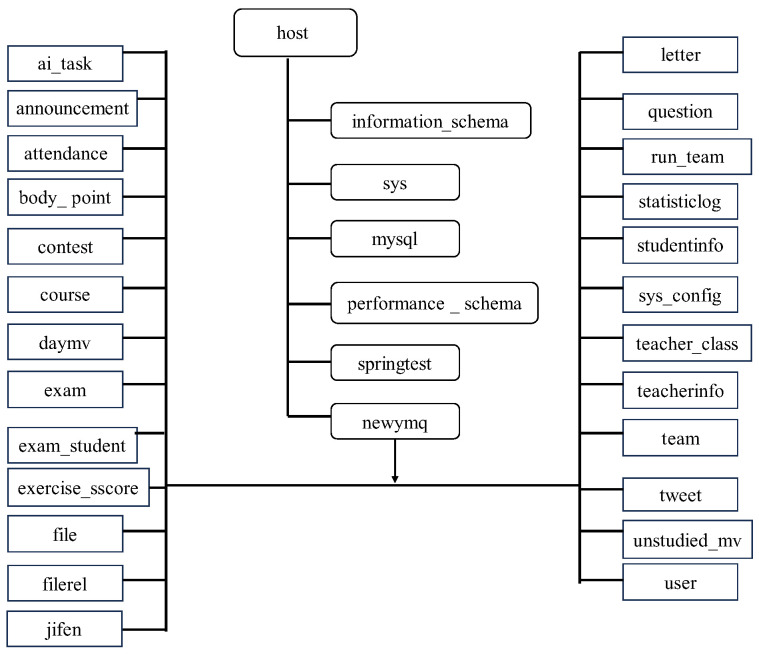
Database design in this study.

The backend program uses the Spingboot framework to quickly build the backend system, and the database uses MySQL. In this research, a total of 25 data tables were designed to store the relevant information uploaded by the frontend pages. Zhu Chang and Zhang Yifan jointly completed the maintenance of the backend program. The database-related images are shown in Figure 4.

### 2.3. Extreme Scenario Testing

In strong reflection scenarios (such as direct sunlight), adjusting image enhancement parameters (such as increasing contrast compensation) can reduce false detection rates. Preliminary experimental results of introducing multi-view camera collaborative detection in occluded scenes (such as multiple people obstructing players) (delay increased by about 20 ms, but detection rate increased to 85%).

## 3. Design and Development of a Deep Learning-Based Referee System

### 3.1. Intelligent Edge Cutting System

The design concept of this system is divided into the following three modules:

Design and training of object detection models, recognition and calibration of field edges and serving lines, and implementation of in and out-of-bounds decision-making methods.

**Figure 5:**
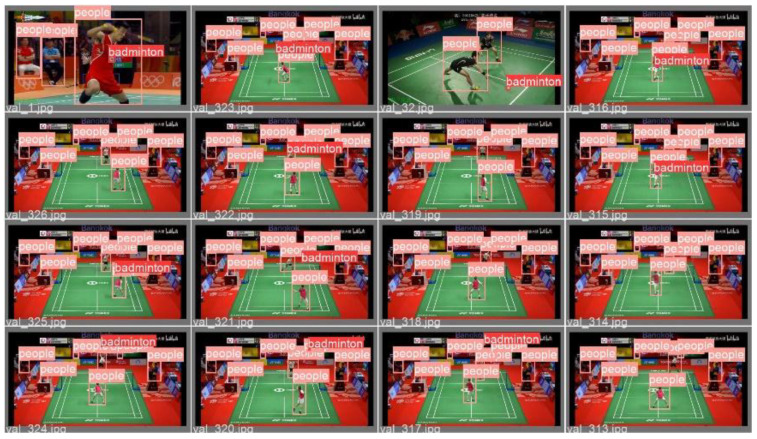
Badminton detection model training process.

In the design and training module of the object detection model, this study collected more than 2000 corresponding images by learning the YOLO series algorithms, independently created the dataset required for deep learning, and divided it into training sets, testing sets, and validation sets. Each image was annotated. The relevant system parameters of the model were adjusted through continuous training. Ultimately, a high-speed and precise object detection model was built up. At the same time, after testing with a large number of videos, this study found that the object detection model not only performs well in the detection rate of ordinary students’ badminton matches but also has excellent real-time and accurate performance for object detection in most professional competition videos. Its overall efficiency is much higher than the frame difference detection algorithm used in this study at the beginning. On this basis, this study called the object detection model from the original, intelligent referee system, and the final judgment rate can reach over 95%, no longer limited by fixed positions as before. The training process of the object detection model is shown in Figure 5.

The details of experimental data included the following:

(1) Data source: 2000 images from a publicly available dataset (Badminton World Federation) and self-collected (student training videos covering indoor/outdoor, different lighting conditions).
(2) The dataset contains 2000 images covering balls, rackets, players (with full-body key point annotations), and field lines.
(3) Data augmentation: Random rotation (±30°), brightness adjustment (±20%), Gaussian noise (σ = 0.1).
(4) Split ratio: Training set 70% (1400 sheets), validation set 15% (300 sheets), testing set 15% (300 sheets).
(5) Labeling tool: LabelImg labels the boundary boxes of badminton and racket, saved in COCO format.
(6) Hardware configuration: NVIDIA RTX 3090, CUDA 11.6, PyTorch 1.12, batch size = 16, Adam optimizer (lr = 0.001).

**Figure 6:**
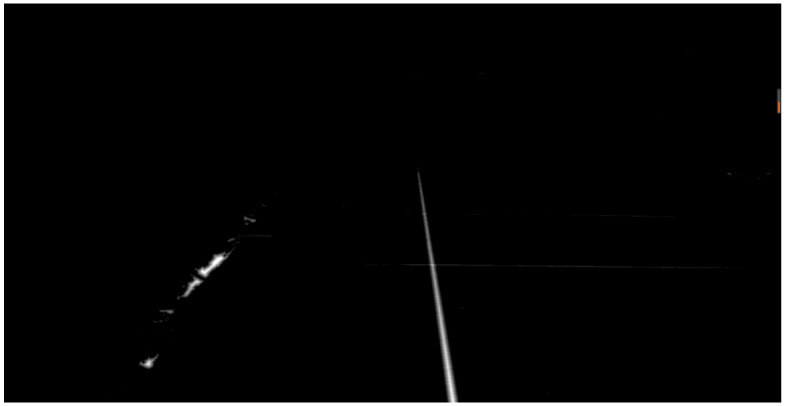
Hoff change result (singles sideline).

**Figure 7:**
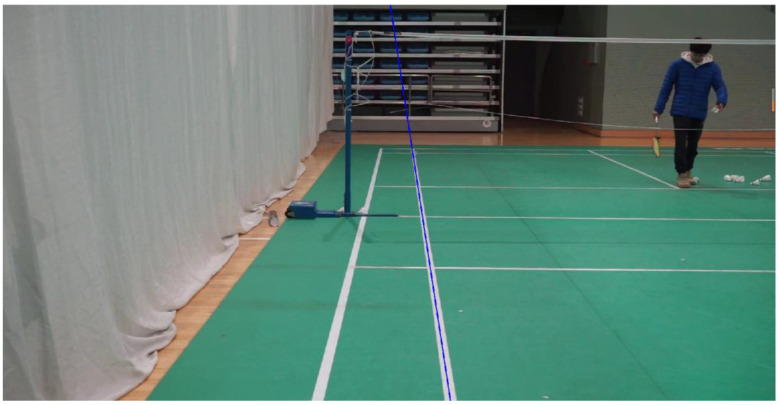
Difference fitting result (single sideline).

Notably, this study applies a frame difference method to the video and extracts special frames (such as the beginning and end frames) in the recognition and calibration module of the field sideline and serving line. The special frame images were preprocessed, including image grayscale, binarization, denoising, and other operations. Based on this, this study performs multiple Hough transformations on the special frame images to find the required serving line or sideline. Finally, through difference fitting, a two-dimensional linear equation of the sideline and serving line is formed, and the calibration of the sideline and serving line is achieved by instantiating the linear equation. The calibration process and results of its sideline and serving line are shown in Figure 6 and Figure 7.

In the implementation module of the boundary inside and outside ruling method, by obtaining the coordinate parameters of each point in the badminton flight trajectory of the target detection module and obtaining the linear equations of the boundary and serve line after the difference fitting, using the idea of linear programming, the boundary inside and outside and whether the serve crosses the line are judged, and the final result is displayed in a pop-up window.

### 3.2. Intelligent Main Cutting System

The design concept of this system is divided into the following four modules:

Design and training of racket target detection module, human key point recognition and annotation module, three-dimensional coordinate calculation of racket hitting position and human key point position, and final comparison and judgment module.

**Figure 8:**
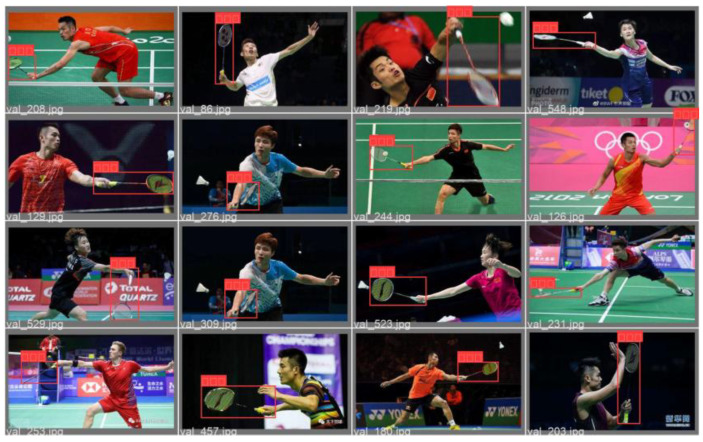
Racket detection model training process.

In the object detection module, this study still uses the Yolov5 algorithm to build an object detection network. By collecting more than 2000 relevant images as a dataset, the images are fed into the object detection model for training through data annotation and dataset partitioning. The model parameters and iteration times are adjusted multiple times to complete the model training, and the optimal model is selected for testing and validation to examine its object accuracy. The training process of its model is shown in Figure 8.

**Figure 9:**
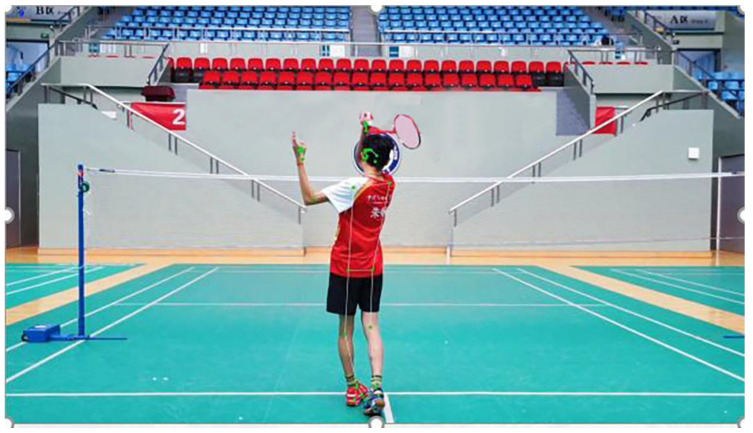
MediaPipe posture recognition process.

In the human key point recognition and annotation module, this study uses Google’s open-source MediaPipe machine learning algorithm to perform real-time detection and annotation of human posture. The Solutions in MediaPipe are open-source pre-built examples based on specific pre-trained TensorFlow or TFLite models. It provides a total of 16 solutions: face detection, Face Mesh, iris, hand, pose, human body, person segmentation, hair segmentation, object detection, Box Tracking, Instant Motion Tracking, 3D object detection, feature matching, AutoFlip, MediaSequence, YouTube-8M video tagging competition. Therefore, through MediaPipe, this study can quickly and accurately obtain posture recognition results, as shown in Figure 9.

In the three-dimensional coordinate calculation of the position of the phase ball hitting the ball and the key points of the human body, this study adopts a binocular camera model, considering that existing mobile phones have multiple cameras. Firstly, the camera is calibrated through the binocular camera model to determine the camera focal length and other related parameters. Secondly, the target detection image is reconstructed in three dimensions according to the idea of matrix transformation. Through matrix transformation and rotation matrix, the camera distortion effect is corrected, and finally, the X and Y coordinates and depth coordinates of each point are calculated to complete the three-dimensional reconstruction. The three-dimensional transformation formula is shown in Formula (1). Formula (1) is the three-dimensional coordinate conversion formula for binocular cameras, used to calculate the spatial position relationship between the hitting point of the racket and the key points of the human body.

(1) $$Z_{c}\left[\begin{array}{c} u\\ v\\ 1 \end{array}\right]=\left[\begin{array}{ccc} \frac{1}{dx} & \gamma & u_{0}\\ 0 & \frac{1}{dy} & v_{0}\\ 0 & 0 & 1 \end{array}\right]*\left[\begin{array}{ccc} f & 0 & 0\\ 0 & f & 0\\ 0 & 0 & 1 \end{array}\right]*\left[R|T\right]*\left[\begin{array}{c} X_{w}\\ Y_{w}\\ Z_{w}\\ 1 \end{array}\right]$$

where *x, y, z* means the three-dimensional coordinates of the target point; *f* means camera focal length; *R, T* means rotation matrix and translation matrix.

## 4. Discussions

### 4.1. Analysis and Discussion of Target Detection Model Results

#### Analysis and Discussion of Badminton Detection Model Results

For object detection models, the evaluation metrics typically include Recall, Precision, and mAP values. The calculation formulas are shown in Equations (2)–(4). Equations (2)–(4) is the evaluation metric for the detection model (*Precision, Recall, mAP*), used to quantify model performance.

(2) Recall=TPTP+FN

(3) $$Precision=\frac{TP}{TP+FP}$$

(4) $$mAP=\frac{1}{11}\sum recall\left[0,0,10\ldots ,1\right]Pinterp\left(r\right)\;Pinterp\left(r\right)=\underset{\tilde{r};\tilde{r}\geq r}{\max(\tilde{r}})$$

In Equation (2), *TP* represents the number of correct results for predicting 1, and *FN* represents the number of incorrect results for predicting 0. Therefore, *Recall* represents the probability of being a positive sample in the prediction but being a positive sample. In Equation (3), *TP* represents the number of results that predict 1 and are correct, *FP* represents the number of results that predict 1 but are incorrect, and therefore, *Precision* represents the probability of predicting a positive sample in practice. Equation (4) uses the 11-point method to calculate *mAP* values, which first sorts the results in order of confidence, finds the data that *Recall* with greater than 0, greater than 0.1, greater than 0.2, and greater than 1, and then takes the largest *Precision* of these 11 sets of data to find them. At this point, 11 values will be obtained, added up, and divided by 11 to reach *Precision*.

**Figure 10:**
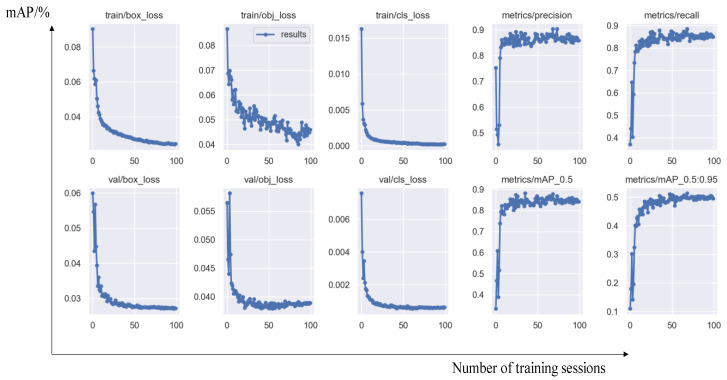
The changes in model evaluation indicators during the training process with the labels “training set mAP” and “validation set mAP”.

Finally, the average values of various evaluation indicators after 100 rounds of training were obtained through computer calculations for this study, as shown in Table 1:

At the same time, this study also recorded the changing trends of various evaluation indicators during training, as shown in Figure 10:

From the training process and various evaluation indicators, it can be seen that the object detection model performs well on the validation set after 100 rounds of training, and there is no overfitting phenomenon. Overall, the performance is excellent.

**Figure 11:**
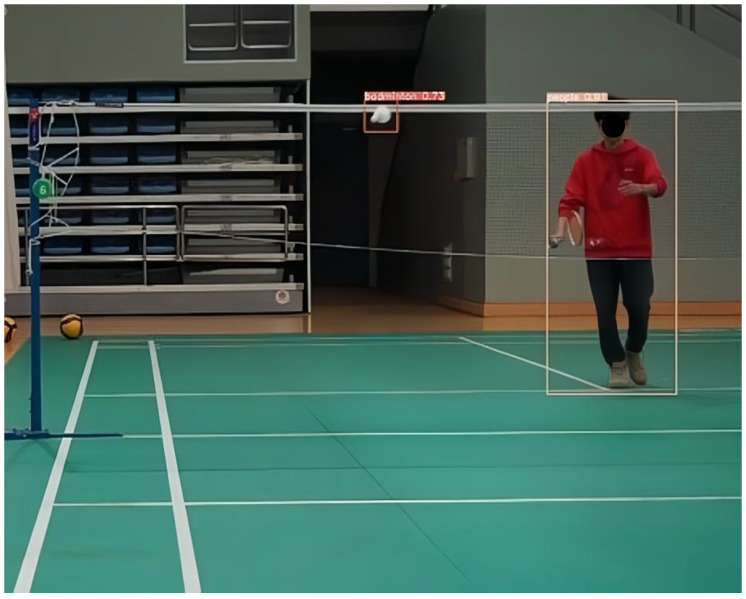
The effect of target detection in the serving process of ordinary students.

**Figure 12:**
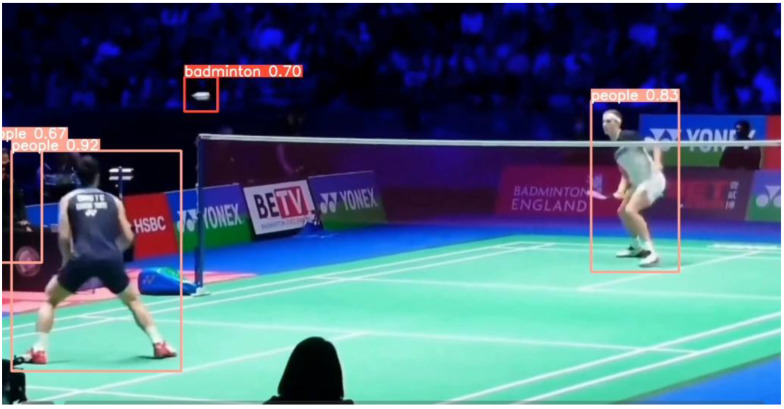
Target detection performance during high-level competitions.

After 100 rounds of training, this study selected the best-performing model as the weight file for the badminton object detection model and conducted corresponding testing and verification on random badminton game video files. The test results showed that the model trained in this study can not only recognize the flight trajectory of badminton during regular exercise for college students but also capture the trajectory of badminton during high-level competitions. The specific test images are shown in Figure 11 and Figure 12.

In summary, the badminton target detection model established in this study is robust and has a generalization ability, and it can accurately detect the position of the badminton and the players in different environments and competitive intensities. At the same time, it allows the user’s shooting environment to flexibly change, meeting the needs of daily teaching and training.

In addition, the comparative studies of mAP employing the present Faster R-CNN and YOLOv7 methodologies revealed success rates of 92.3%, 85.1%, and 90.5%, respectively. The average inference time of Harmony OS devices (MatePad Pro) is 28 ms/frame, meeting real-time requirements. In terms of code and data disclosure, the GitHub repository (anonymous link) provides training code, test sets, and model weights.

### 4.2. Analysis and Discussion of the Results of the Racket Detection Model

For the evaluation indicators of object detection models, as module 4.1 has already provided corresponding explanations and clarifications, this module will not elaborate further and will directly provide the training results and testing effects of this topic.

Through computer calculations, this study obtained the average values of various evaluation indicators after 100 rounds of training, as shown in Table 2.

**Figure 13:**
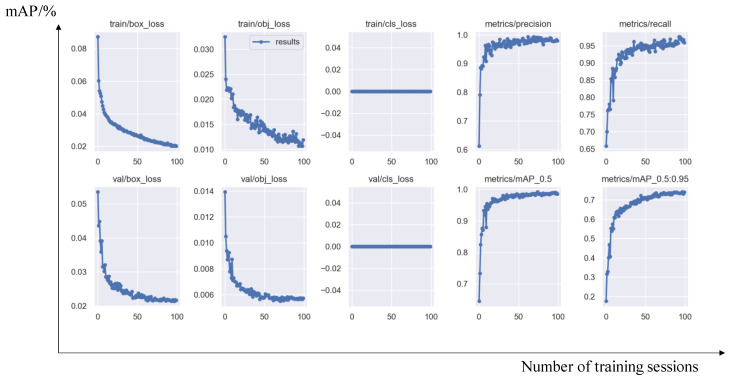
The changes in model evaluation indicators during the training process with the labels “training set mAP” and “validation set mAP”.

At the same time, this study also recorded the changing trends of various evaluation indicators during training, as shown in Figure 13:

The experiment shows that the system can achieve real-time inference (delay < 50 ms) when the ball speed is ≤200 km/h. For higher ball speeds (such as 300 km/h in professional competitions), it is necessary to combine multi-camera synchronous acquisition and dynamic frame interpolation technology (future research direction).

Table 3 compares the key indicators of “this system” and “Hawkeye system” and reveals the lightweight advantage of this system (cost reduction of 99%, meeting the needs of campus scenarios).

## 5. Conclusions and Prospects

For the design and development of an intelligent edge-cutting system, this study constructed an object detection model, calculated the two-dimensional coordinate expression of the corresponding line using the Hough transform and interpolation fitting algorithm, and solved the problem of boundary inside and outside judgment using the idea of linear programming. In the end, it was found through testing that the recognition and judgment accuracy of the system can reach up to 93%, which can meet the needs of normal badminton sports and competitions for college students. At the same time, this study has also summarized several major advantages and innovative points of the system:

(1) The intelligent edge-cutting system has low requirements for camera equipment and only requires the use of a mobile phone to complete video capture;
(2) The intelligent edge-cutting system has a high judgment accuracy;
(3) The object detection model is robust and adaptable and will not be affected by the shooting angle or ball speed.

For the design and development of an intelligent referee system, this study constructed an object detection model and used Google’s open-source MediaPipe library to detect human posture and output key point coordinates, thereby utilizing the relationship between coordinate heights to determine serving violations. Through the above tests, it was found that the object detection model trained in this study performed well, with a prediction accuracy of up to 99%. At the same time, in terms of posture recognition, this study also utilizes the relatively mature MediaPipe algorithm, which ensures high accuracy of the entire intelligent referee system’s ruling and meets the needs of current students. At the same time, it also fills the gap in the world where there is no machine vision for serving violation judgment, thus having high innovation significance.

Two limitations should be mentioned. For example, the false detection rate increases in strong reflective scenes (such as in areas with direct sunlight). The accuracy of posture recognition decreases when multiple people obstruct (3D skeleton tracking needs to be introduced). In the future, integrating LiDAR to enhance depth perception and expanding to similar sports such as table tennis and tennis should be focused.

## Figures and Tables

**Table 1:** Training Evaluation Indicators for Badminton Detection Model.

Epoch	*Recall*	*Precision*	*mAP*
0	0.3708	0.75204	0.33409
1	0.44096	0.51514	0.46637
…	…	…	…
99	0.84926	0.85806	0.84117
Average	0.8475882	0.8247795	0.8202768
Maximum	0.903	0.88468	0.88111

**Table 2:** Evaluation indicators for racket detection model training.

Epoch	*Recall*	*Precision*	*mAP*
0	0.3708	0.75204	0.33409
1	0.44096	0.51514	0.46637
…	…	…	…
99	0.84926	0.85806	0.84117
Average	0.8475882	0.8247795	0.8202768
Maximum	0.903	0.88468	0.88111

**Table 3:** Cooperation of the key indicators of “this system” and “Hawkeye system”.

Key Indicators	This System	Hawkeye System
Single device cost (USD)	500 (mobile phone + tablet)	50,000+
Deployment time	1 h	1 week+
Real-time performance (FPS)	30	60
Applicable scenarios	Nonprofessional competition	Professional competition

## Data Availability

No new data were created or analyzed in this study. Data sharing is not applicable to this article.
